# Sialic acid: a sweet swing between mammalian host and *Trypanosoma cruzi*

**DOI:** 10.3389/fimmu.2012.00356

**Published:** 2012-11-29

**Authors:** Leonardo Freire-de-Lima, Isadora A. Oliveira, Jorge L. Neves, Luciana L. Penha, Frederico Alisson-Silva, Wagner B. Dias, Adriane R. Todeschini

**Affiliations:** Laboratório de Glicobiologia Estrutural e Funcional, Instituto de Biofísica Carlos Chagas Filho, Universidade Federal do Rio de Janeiro, Rio de JaneiroBrazil

**Keywords:** glycoconjugate, sialic acid, sialidase, parasite, immune response

## Abstract

Commonly found at the outermost ends of complex carbohydrates in extracellular medium or on outer cell membranes, sialic acids play important roles in a myriad of biological processes. Mammals synthesize sialic acid through a complex pathway, but *Trypanosoma cruzi*, the agent of Chagas’ disease, evolved to obtain sialic acid from its host through a *trans*-sialidase (TcTS) reaction. Studies of the parasite cell surface architecture and biochemistry indicate that a unique system comprising sialoglycoproteins and sialyl-binding proteins assists the parasite in several functions including parasite survival, infectivity, and host–cell recognition. Additionally, TcTS activity is capable of extensively remodeling host cell glycomolecules, playing a role as virulence factor. This review presents the state of the art of parasite sialobiology, highlighting how the interplay between host and parasite sialic acid helps the pathogen to evade host defense mechanisms and ensure lifetime host parasitism.

## THE SIALIC ACIDS

**FIGURE 1:**
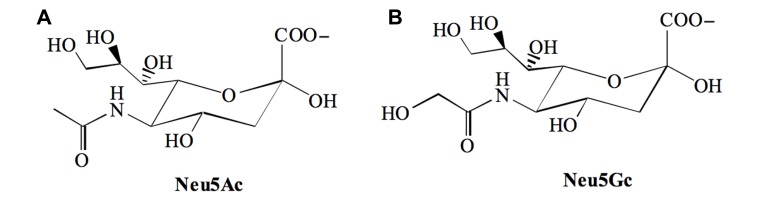
**The two major sialic acid found in mammals**. **(A)** *N*-acetylneuraminic acid [Neu5Ac] and, **(B)** *N*-glycolylneuraminic acid [Neu5Gc].

Sialic acids (Sia) are 9-carbon backbone acidic monosaccharides found at prominent positions of the sugar chains of glycoconjugates present on cell membranes or secreted into the extracellular medium. The most common members of this family are the *N*-acetylneuraminic acid [Neu5Ac] and its derivative the *N*-glycolylneuraminic acid [Neu5Gc] that differ from each other at position 5 (C-5), which is substituted with an acetamido or a hydroxyacetamido moiety respectively (**Figure 1**).

The metabolism of Sia in mammals involves 32 genes that encode enzymes and transporters, distributed among the different compartments of the cell (Wickramasinghe and Medrano, 2011). The final product of this complex biosynthetic pathway, the activated form of Sia (CMP-Sia), is transferred to the non-reducing end of newly synthesized glycan chains by a family of sialyltransferases present in the Golgi lumen. In vertebrates, Sia are commonly linked via an α2–3 linkage to galactopyranose (Gal*p*), via an α2–6 linkage to Gal*p* and *N*-acetylgalactosamine (GalNAc), or via an α2–8 linkage to another Sia (Varki et al., 2009).

Sia-containing glycoconjugates are involved in a myriad of cell functions. Because of the negative charge, these molecules affect the recognition and anti-recognition phenomena. They are targets of Sia-binding lectins and can mask underlying structures, for example impeding the binding of Gal-specific receptors (Sørensen et al., 2009; Rabinovich et al., 2012).

Sia are also important as recognition sites in host–pathogen interactions acting as ligands for parasite adherence, possibly driving natural selection (Varki, 2006). Further, Sia play a major role protecting the infective agent from the host’s immune response through molecular mimicry while unsialylated strains are rapidly cleared. The evolutionary advantage of this molecular mimicry is so evident that several parasites exploit cell-surface sialylation to survive within the host environment and establish the infection (Vimr and Lichtensteiger, 2002).

**FIGURE 2:**
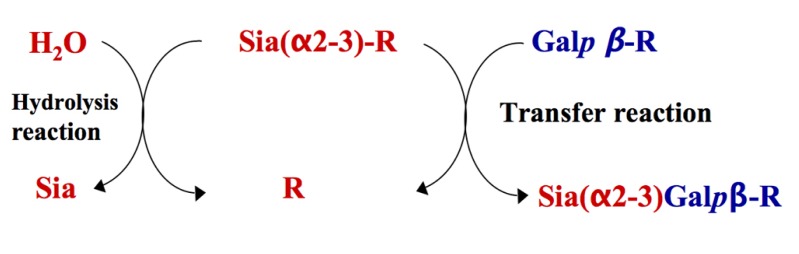
**Representation of transfer and hydrolysis activities of TcTS**. TcTS preferentially catalyzes the transfer of sialic acid residues from Siaα2–3Galβ1-R containing donors and attaches them in α2–3 linkage to terminal β-Gal*p* containing acceptors (transfer reaction). In the absence of a carbohydrate acceptor, TcTS irreversibly transfers sialic acid to a water molecule, thus functioning as a sialidase (hydrolysis reaction).

One of the most elegant mechanisms of cell-surface sialylation is exploited by the protozoal parasite *Trypanosoma cruzi*, the etiological agent of Chagas’ disease (Coura and Viñas, 2010). *T. cruzi* is not able to synthesize Sia. Instead the parasite scavenges it from host’s sialyl glycoconjugates using its *trans*-sialidase activity (TcTS; Previato et al., 1985). TcTS is a modified sialidase, which preferentially transfers α2-3-linked Sia from sialyl β-Gal*p* donor complexes of exogenous origin to acceptor surface mucin-like glycoproteins containing terminal β-galactopyranose residues (**Figure 2**). In the absence of suitable acceptors TcTS transfers Sia to water, then acting as a sialidase (Vandekerckhove et al., 1992).

**FIGURE 3:**
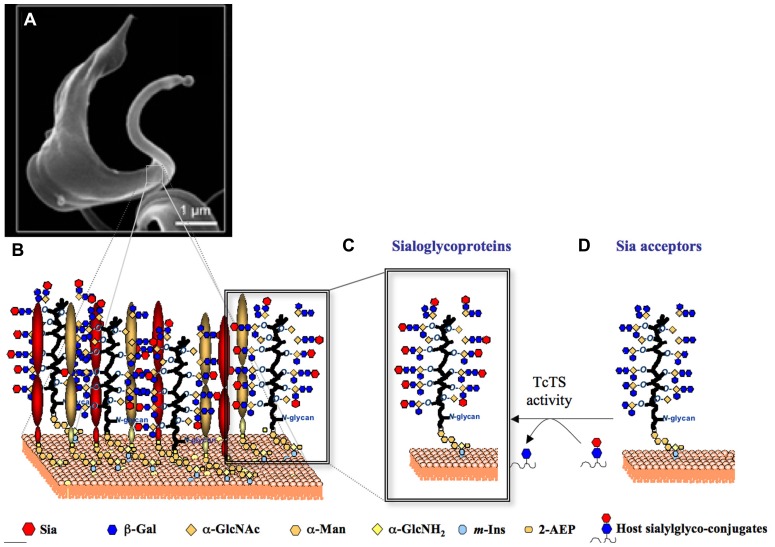
**Representation of the TcTS/sialoglycoproteins system on *T. cruzi* trypomastigotes surface**. **(A)** A scanning electron micrograph of a bloodstream form *T. cruzi* trypomastigote (www.fiocruz.br/chagas_eng/cgi/cgilua.exe/sys/start.htm?sid=13) is shown. **(B)** The cartoon represents the GPI-anchored TcTS, TcTS_Y342H_/sialoglycoproteins covering the trypomastigote surface. Sia present on *T. cruzi* sialoglycoproteins **(C)** is transfered from host sialoglycoconjugates to β-Gal*p* containing glycoproteins expressed on parasite surface **(D)** by TcTS activity.

Once restricted to Latin America, where it affects ~10 million people, Chaga’s disease become a new worldwide challenge. It has now spread to North America, Europe, and the western Pacific region (Chappuis et al., 2010; Clayton, 2010). Molecules of the TcTS family (Freitas et al., 2011) and Sia acceptor glycoproteins (De Pablos and Osuna, 2012) are encoded by hundreds of genes. Combined, the TcTS and the mucin-like glycoproteins are likely to cover most of the parasite surface (**Figure 3B**) creating a parasite–host interface (**Figure 3A**)

In this review we discuss the importance of this unique biological system, comprised by both TcTS and mucin-like Sia acceptor glycoproteins, highlighting how the parasite explores host cell sialylation to establish infection for the lifetime of its host.

## THE SIALIC ACID ACCEPTORS ON THE SURFACE OF THE PARASITE AND ITS ROLE IN HOST PARASITE INTERACTION

The Sia acceptors on the surface of *T. cruzi *are mainly a family of highly *O*-glycosylated, threonine-rich mucin like glycoproteins (**Figure 3D**; Buscaglia et al., 2006; Mendoncc, 2008) which are glycosylphosphatidylinositol (GPI)-anchored to the parasite membrane (Previato et al., 1995). The Tc-mucins are the major expressed component on the of *T*. *cruzi *(2 × 10^6^ copies per parasite) and are the third most widely expanded gene family in the genome, comprising more than 1000 genes (Acosta-Serrano et al., 2001; Campo et al., 2004; El-Sayed et al., 2005; De Pablos and Osuna, 2012). Carrying up to 60% of its total mass in carbohydrates, mucins form an elaborate and highly decorated glycocalyx that allows the parasite to interact with and respond to its external environment. Furthermore, glycoproteins expressed on the parasite surface are known to be the major targets of protective immune responses, and this selective pressure presumably drives their expansion and variation among different strains (Minning et al., 2011).

The glycan structure of mucin is complex and heterogeneous among different *T. cruzi* strains. The structure of the oligosaccharides *O*-linked to the mucins of the non-infective epimastigote forms have been described (Previato et al., 1994, 1995; Todeschini et al., 2001, 2009; Agrellos et al., 2003; Jones et al., 2004). These *O*-glycans differ from those found in mammalian systems in three main aspects: (i) they are linked to the peptide backbone through an α-*N*-acetylglucosamine (α-GlcNAc) residue (Previato et al., 1998), rather than an α-*N*-acetylgalactosamine (α-GalNAc; Gill et al., 2011); (ii) they are further substituted by (Gal) on *O*-4 and *O*-6 rather than *O*-3 and *O*-6 as found in mammalian mucins (Varki et al., 2009); (iii) several strains carry a β-galactofuranose (β-Gal*f*) attached to the GlcNAc *O*-4 (Previato et al., 1994; Agrellos et al., 2003; Jones et al., 2004). Two core families were characterized with substitution of the α-GlcNAc *O*-4 by either β-Gal*p *or β-Gal*f*. The Gal*f*β1-4GlcNAc core can be further elaborated by the action of galactopyranosyl or galactofuranosyl transferases. Additionally, substitution at the GlcNAc-ol *O*-6 can occur. Addition of β-Gal*f* to mucins must be verified given that *T. cruzi* presents dozens β-galactofuranosyl transferase genes (El-Sayed et al., 2005; De Pablos and Osuna, 2012) while mammalian hosts glycoconjugates do not present this modification. β-Gal*f* residues might account for host parasite selection since the presence of β-Gal*f*-containing mucins are expressed mainly by strains involved in the sylvatic cycle of *T. cruzi* (Jones et al., 2004).

The major sialylated oligosaccharides (**Figure 3C**) so far characterized comprise a Neu5Acα2-3Gal*p*β1-4GlcNAc (Jones et al., 2004), a Gal*f*β1-4(Neu5Acα2-3Gal*p*β16)GlcNAc (Agrellos et al., 2003), a Gal*p*β1-4(Neu5Acα2-3Gal*p*β1-6)GlcNAc, and a Gal*p*β1-6(Neu5Acα2-3Gal*p*β(1-4)GlcNAc (Todeschini et al., 2001). Characterization of the related monosialylated glycans confirms a previous report showing that incorporation of one Sia residue onto an acceptor hinders entry of a second residue (Previato et al., 1995). Thus, it is possible that concomitant presence of both α2-3-linked Sia and terminal β-Gal*p* residues have biological implications in the *T. cruzi*/host cell interaction.

In addition to the *O*-glycosylation observed in the mucins of epimastigote forms, mucin glycans of infective trypomastigotes derived from infected mammalian cells also contain terminal α-galactosyl residues. α-Gal containing saccharides are epitopes recognized by lytic antibodies found in chronic Chagasic patients (Almeida et al., 1994). Sialylation of parasite glycoconjugates confers significant resistance to killing by the lytic antibodies, which is in agreement with the hypothesis that sialylation must favor parasite survival (Pereira-Chioccola et al., 2000). Further evidence corroborating this hypothesis is that the presence of Sias on the parasite mucins may compromise the activation of the complement pathway (Tomlinson et al., 1994). After sialidase treatment, parasites become more sensitive to complement-induced lysis. Also, parasite uptake by macrophages is increased (Tomlinson et al., 1994).

The importance of Sia on the parasite surface during host cell infection is still controversial. While some studies have shown that the presence of Sias in parasite epitopes increases *T. cruzi* infection (Piras et al., 1987; Schenkman et al., 1991), other groups suggest that the presence of Sias is not a requirement and/or may compromise the invasion of host cells (AraÚjo-Jorge and De Souza, 1988; Yoshida et al., 1997).

Finally, there are increasing evidences to support the Sia-binding Ig-like lectin (Siglecs), on the host cell surface, as the coreceptor for *T. cruzi* mucin (Erdmann et al., 2009; Jacobs et al., 2010). The Siglecs are a family of sialic-acid-binding immunoglobulin-like lectins that promote cell–cell interactions and regulate the functions of cells in the innate and adaptive immune systems through glycan recognition (Varki and Gagneux, 2012). It was demonstrated that *T. cruzi* mucin engagement with the Sia-binding protein Siglec-E promotes immunosuppression of dendritic cells (DC; Erdmann et al., 2009).

## TcTS ACTIVITY AND ITS ROLE IN HOST PARASITE INTERACTION

TcTS is part of a protein family known as trans-sialidase/trans-sialidase-like, encoded by more than 1,400 genes (El-Sayed et al., 2005; Freitas et al., 2011). Members of the TcTS gene family can be classified in five groups based on sequence similarity and functional properties (Freitas et al., 2011). Active TS, also namely SAPA (shed acute-phase antigen) expressed by the infective trypomastigote (tTS) and the epimastigote TS (eTS) are grouped into Group I. eTS and tTS have identical enzymatic activities, being highly conserved in their primary sequences (Chaves et al., 1993; Briones et al., 1995; Jager et al., 2008), except for the SAPA domain and their 3′ UTRs, which are completely different in sequence (Jager et al., 2007). Besides, eTS is a trans-membrane protein, while the tTS is associated with the membrane via a GPI linker (Agusti et al., 1997). Group II comprises members of the gp85 surface glycoproteins TSA-1, SA85, gp90, gp82, and ASP-2, which have been implicated in host cell attachment and invasion. FL-160, a representative of group III, is a complementary regulatory protein that inhibits the alternative and classical complement pathways. TsTc13, whose function is unknown, is the representative of group IV and is included in the TcS superfamily because it contains the conserved VTVxNVxL (Alves and Colli, 2008; Yoshida and Cortez, 2008; Souza et al., 2010). Recently a sequence clustering analysis demonstrated that TS family is even more complex and may arbor more groups and subgroups (Freitas et al., 2011).

Several studies suggest that the TcTS can sialylate or desialylate host cells modulating parasite adherence and penetration. Results with Sia-deficient mutants of Chinese hamster ovary (CHO) cells support this hypothesis (Ciavaglia et al., 1993; Ming et al., 1993). Sia deficient cells were less infected than wild-type cells, suggesting that sialylation of glycoconjugates on CHO cells surface is necessary during *T. cruzi* invasion. Moreover, treatment of cells with modified Sia precursors, *N*-propionylmannosamine and other *N*-acylmannosamines, decreased cell invasion by *T. cruzi *(Lieke et al., 2011). Importance of TcTS enzymatic activity in host cell invasion was elegantly proven using an irreversible inhibitor (Carvalho et al., 2010a).

On the other hand, desialylation of sialoglycoproteins found in the membrane of phagolysosomes by TcTS is thought to be important for the escape of the parasite from the cytoplasm of infected cells (Hall et al., 1992; Hall and Joiner, 1993; Rubin-de-Celis et al., 2006).

Glycosylphosphatidylinositol-linked trypomastigote-derived TcTS can be released into the extracellular medium in fairly high amounts during acute *T. cruzi* infection in humans, thus acting distant from the parasite as a soluble factor. Besides its role in mammalian cell invasion, the soluble form of TcTS functions as a virulence determinant molecule, and therefore, could have relevant biological effects on the host immune system. It has been demonstrated (Chuenkova and Pereira, 1995) that *in vivo* injection of tiny amounts of purified native TcTS increases subsequent parasitemia and mortality in *T. cruzi*-infected mice. The effect observed was specific for the transfer activity of TcTS because it did not occur in mice primed with viral or bacterial sialidases. The mechanisms responsible for these effects were not determined but, since TcTS injection into deficient SCID mice did not affect parasitemia or mortality, it was suggested that the enzyme acts on host lymphocytes of the acquired immune system (Chuenkova and Pereira, 1995).

Indeed, multiple effects of TcTS on host T-lymphocyte function were additionally demonstrated. TcTS engagement with α2-3-linked Sia-containing epitopes on CD43 (Todeschini et al., 2002a) from CD4^+^ T cells triggers costimulatory responses that increase mitogenesis and cytokine secretion, as well as promote rescue from apoptosis (Todeschini et al., 2002b). These results strongly suggest that TcTS could be a key parasite molecule inducing host polyclonal lymphocyte activation, seen as a condition underlying induction of immunopathology and hampering effective vaccination (Minoprio et al., 1989) in the course of *T. cruzi* infection.

Given that surface sialylation might be crucial to decide the final fate of the cells during interaction with thymic lectins (Gillespie et al., 1993; Priatel et al., 2000) alteration of cell sialylation by the soluble TcTS might influence thymocyte development. In fact, alteration of the surface sialylation by TcTS (Mucci et al., 2006) leads to *in vivo* depletion of the CD4^+^CD8^+^ double-positive thymocytes inside the “nurse cell complex” (Leguizamón et al., 1999). Interestingly, thymocyte apoptosis observed after the sialyl residue mobilization requires the presence of androgens (Mucci et al., 2005), suggesting the presence of a dimorphic glycosylation survey in the development of the T cell compartment that can be related to the observed differences in the immune response among sexes (Gui et al., 2012). However, further studies about the molecular mechanism involved in the pro-apoptotic effect of TcTS are necessary. However, we can speculate that TcTS activity can mask or expose β-Gal*p* which is recognized by molecules of the galectin family. Corroborating this hypothesis a role was reported for galectin-3 in death of CD4^+^CD8^+^ immature thymocytes and migration of these cells away from the thymus after *T. cruzi* infection (Silva-Monteiro et al., 2007).

**FIGURE 4:**
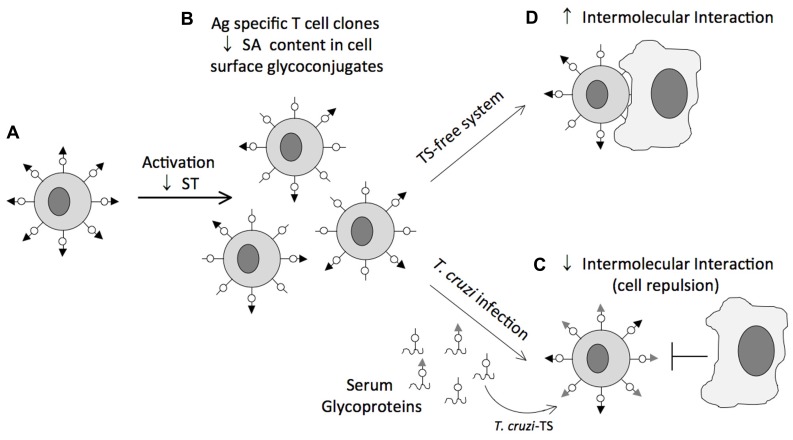
**Representative scheme of the glycosylation status of CD8^+^ T cells during *T. cruzi* infection**. Naïve CD8^+^ T cells are heavily sialylated **(A)**. During T cell activation, down-regulation of sialyltransferases (ST) renders potential sialic acid acceptors accessible to sialylation through TcTS activity **(B)**. This sialylation may be advantageous to the parasite, because CD8^+^ T cells resialylated by TcTS present compromised Ag-specific responses since sialic acid charge might increases intercellular repulsion and therefore weakens TCR/MHC class I-mediated cell–cell interactions **(C)**. Absence of TS may favor the TCR/MHC class I cell–cell interaction consequently, development of the immune response **(D)**.

The impact of sialylation mediated by TcTS on CD8^+^ T cell response of mice infected with *T*. *cruzi* is an exciting example of how a parasite can manipulate host cell sialylation to favor parasitism. Following infection CD8^+^ T cell responses are robust and persistent. However, they are significantly delayed (Garg et al., 1997; Tzelepis et al., 2007). This delay contrasts with the rapid appearance of CD8^+^ T cell responses in other viral, bacterial and even protozoal infections (Kaech et al., 2002), and suggests a operative mechanism of immune evasion. During T cell activation, down-regulation of sialyltransferases (Amado et al., 2004) renders potential Sia acceptors accessible to sialylation through TcTS activity (**Figure 4**). This sialylation may be advantageous to the parasite, since CD8^+^ T cells resialylated by TcTS present compromised Ag-specific responses and TcTS-treated mice present increased parasitemia (Freire-de-Lima et al., 2010). Cell surface Sia on CD8^+^ T cells might increase intercellular repulsion and therefore weaken TCR/MHC class I-mediated cell–cell interactions. This would be the opposite of the effect of neuraminidase treatment, which removes Sia residues from various membrane glycoproteins and enhances lymphocyte proliferation (Harrington et al., 2000). In an attempt to establish the nature of the Sia acceptor for TcTS on the CD8^+^ T cell surface, CD8^+^ T cells from mice lacking the ST3Gal-I sialyltransferase, an enzyme required for sialylation of core 1 *O*-glycans (Priatel et al., 2000), were infected with *T. cruzi*. Loss of ST3Gal-I sialyltransferase exposes the Galβ1-3GalNAc-Ser/Thr moiety creating an interesting model to establish CD43 as a natural receptor for native TcTS during *T. cruzi* infection. Indeed, infection of mice lacking ST3-Gal-I sialyltransferase restores, at least in part, binding of anti-CD43 S7 mAb, which recognizes Sia-containing epitopes on CD43 of CD8^+^ T cells. These findings indicated that CD43 is a target receptor for TS on the CD8^+^ T cell surface. However, resialylation by TcTS was also observed on CD8^+^ T cells from CD43 KO mice, suggesting that in the absence of CD43 other molecules are substrates for TcTS. Other studies using azido-modified unnatural Sia revealed that CD45 isoforms are Sia acceptors for TcTS activity as well (Muiá et al., 2010).

In infected individuals, alteration of cell surface sialylation by TcTS can also compromise host cell homeostasis. Tribulatti et al. (2005) demonstrated that the administration of TcTS into uninfected mice was able to reduce the Sia content of platelets, exposing terminal galactose residues, which may explain the severe thrombocytopenia observed in *T. cruzi* infected individuals. The recognition of terminal galactose moiety exposed on the platelet surface accelerates platelet clearance by asialoglycoprotein receptor-expressing scavenger cells (Sørensen et al., 2009). The effect of TcTS on the lifetime of other cell types and plasma glycoproteins must be further verified.

Beyond host immune response, it has been observed that TcTS alters the sialylation status of the tyrosine kinase receptor-A (TrkA) in PC12 cells, which leads to receptor internalization, activation, and neuronal differentiation (Woronowicz et al., 2004). Authors demonstrated that the effects observed are triggered by hydrolysis of Sia residues of TrkA by TcTS, as a purified recombinant α2–3-neuraminidase but not a catalytically inactive mutant of TcTS induced the receptor phosphorylation. Such enzymatic activity might be involved in the neural repair and neuroprotection mediated by the TcTS, also called *T. cruzi*-derived neurotrophic factor (Chuenkova and Pereiraperrin, 2011).

The examples described in this section strongly suggest that *T. cruzi *exploits the glycosylation of molecules expressed by the host to evasion of the immune response, thus perpetuating the infection.

## INACTIVE TcTS

Inactive TcTS (TcTS_Y342H_) is a parasite adhesin that differs from the active TcTS due to a single mutation of catalytic residue Tyr342, which is mainly changed by a histidine (Cremona et al., 1995). In some *T. cruzi* strains, genes encoding TcTS_Y342H_ members are present in the same copy number as those encoding TcTS (Cremona et al., 1999). However, further studies should be performed in order to address the expression levels and the ratio of the TcTS: TcTS_Y342H_ protein on *T. cruzi* surface.

TcTS_Y342H_ is a unique adhesin containing two sugar binding sites: one for α2,3-Sia and other for β-Gal*p* (Todeschini et al., 2002a, 2004; Oppezzo et al., 2011). Interestingly, the carbohydrate recognition domain for β-Gal*p* residue is formed only after a conformational switch triggered by prior sialoside binding (Todeschini et al., 2004). The bivalent nature of TcTS_Y342H_ might promote glycan cross-linking, which is believed to be essential for cellular signal transduction.

The finding that inactive TS has two carbohydrate binding domains, may explain some apparently contradictory results on the involvement of sialyl and galactosyl epitopes in *T. cruzi*/host cell interaction. While Schenkman et al. (1991) have shown that sialylation of Ssp-3 epitope of mammalian cell-derived trypomastigotes is required for target cell recognition, Yoshida et al. (1997) reported that the removal of Sia from the surface of insect-derived metacyclic trypomastigotes enhances parasite-host interaction. The removal of Sia from *T. cruzi* glycoproteins and the concomitant exposure of cryptic β-Gal*p* residues would favor TcTS_Y342H_ interaction with both host sialoglycoconjugates and terminal β-Gal*p*-containing glycoproteins on the parasite surface, thus enhancing *T. cruzi*/host adhesion. This phenomenon was well characterized for CD22, a mammalian Sia-binding lectin (Varki and Gagneux, 2012). The removal of Sia and concomitant exposure of β-Gal*p* residues from host cell glycans, which occurs as a result of the *T. cruzi* TS reaction may, therefore, be physiologically significant by promoting parasite adherence to, and penetration of host cells.

On the other hand, the parasite might use the active TS to sialylate host cell glycomolecules and generate receptors for TcTS_Y342H_ mediating trypanosome adherence to a target cells. Data showing that sialic acid-deficient cells are less infected than wild-type cells (Ciavaglia et al., 1993; Ming et al., 1993), suggest that recognition of sialyl residues on CHO cells by TcTS_Y342H_ is necessary during *T. cruzi* invasion.

The hypothesis that TcTS_Y342H_ promotes glycan cross-linking can be corroborated by data showing that both forms, active and TcTS_Y342H_, bind to α2,3-Sia from CD43 on host CD4^+^ T cells, triggering a co-stimulatory response through mitogen-activated protein kinase ERK1/2 cascade inducing mitogenesis (Todeschini et al., 2002b). It was also shown that both forms of TcTS protect neuronal and glial cells from apoptosis through activation of PI3K/Akt pathway and up-regulate the anti-apoptotic bcl-2 gene (Chuenkova and Pereira, 2000; Chuenkova et al., 2001). Furthermore, we showed that TcTS_Y342H_ binds to α2,3-Sia containing molecules on endothelial cells resulting in NF-κB activation, expression of cell adhesion molecules and rescue from apoptosis. Activation of endothelial cells increases trypomastigotes attachment and invasion, suggesting that TcTS_Y342H_ plays a role in host cell invasion during *T. cruzi* infection (Dias et al., 2008). Further studies are required to establish the overall role of TcTS_Y342H_ in the pathogenesis of Chagas’ disease. Data showing that TcTS_Y342H_ competes *in vivo* with the native TcTS for Sia and β-Gal binding sites, inhibiting potential sialylation events from taking place (Freire-de-Lima et al., 2010), might suggest that TcTS_Y342H_ benefits the host during *T. cruzi* infection. Decreased mortality of mice treated with TcTS_Y342H_ suggests a role in prolonging parasite persistence in host tissues and corroborates the role of TcTS-mediated sialylation in the virulence of *T. cruzi.*

Yet the ability of *T. cruzi* to exploit host glycocalyx to attach and establish the infection must be further studied as other inactive members of TcTS might have lectinic properties. Two motifs common to other sialidases, FRIP (xRxP) and Asp box, can be found in various groups of the *trans*-sialidase-like family (Freitas et al., 2011). The FRIP motif, involved in binding the carboxylate group of sialic acid (Gaskell et al., 1995), is found in four out of seven groups of the *trans*-sialidase-like proteins. The Asp box follows the FRIP motif and can be repeated up to five times in the sequences of viral, bacterial, trypanosomatid, and mammalian sialidases. Although its function is unknown, it is worth noting that the Asp box occurs in secreted proteins and in proteins that act on, or interact with, carbohydrates (Copley et al., 2001). Growing evidence has shown that the insect vector-derived metacyclic trypomastigote uses its stage-specific surface molecule gp82, which is member of the gp85/TS superfamily, to bind to gastric mucin and establish *T. cruzi* infection in mice by the oral route (Neira et al., 2003; Staquicini et al., 2010; Cortez et al., 2012).

Findings showing that TcTS_Y342H_ act as a lectin open a new avenue to be explored in the interaction of *T. cruzi* and its hosts. These studies raise the hypothesis that TcTS_Y342H_ helps the parasite to bind to surfaces rich in sialylated glycoconjugates. In addition, TcTS_Y342H_ can act as a lectin triggering cellular signaling, or helping the TcTS from *T. cruzi* surface to transfer and decorate the cellular surfaces with Sia.

## TcTS MECHANISM

TcTS is a retaining glycoside hydrolase (Todeschini et al., 2000) member of the family number 33 (GH-33) that comprises the bacterial and eukaryotic exo-alpha sialidases (Withers and Williams, 2012). Unlike it’s closely related sialidase, TcTS preferentially transfers sialic acid units to terminal β-Gal*p*-containing molecules and synthesizes α2-3-linkages exclusively. Efforts to decipher the mechanism of TcTS catalysis have been important to the dissection of the mechanism of exo-alpha sialidases (Watts et al., 2006; Newstead et al., 2008) as these enzymes, unlike most of the retaining glycoside hydrolases, do not present a carboxylate correctly placed in the active site to act as a nucleophile. Pioneering studies with fluor-based sugar inactivators (Watts et al., 2003) followed by Lc-MS/MS peptide mapping and then crystallography (Amaya et al., 2004) demonstrated that TcTS operates through a double displacement mechanism involving the transient formation of a covalent sialyl-enzyme intermediate with a Tyrosine (Tyr342), a very conserved residue in exo-sialidases (Vocadlo and Davies, 20083; Davies et al., 2012), while D59 was proposed to act as a general acid/base catalyst.

From such results a classical ping-pong mechanism (Damager et al., 2008) was inferred for TcTS, where the sialosyl aglycone may abandon the active site to allow the entry of acceptor substrate. In this mechanism, as the sialic acid unit approaches the enzyme, it displaces the Tyr119 away from the binding site (Buschiazzo et al., 2002), its carboxylate group interacts with the Arg triad (Arg35, Arg245, Arg314), while its acetamido group interacts with Asp96. Such interactions induce planarization of the sialic acid moiety around the oxygen ring, with C1, C2, and C3 assuming a ^4^H_5_ conformation during the transition state. The C2 suffers nucleophilic attack by Tyr342, assisted by Glu230 acting as a general base, and a covalent linkage is formed. The covalent intermediate assumes a ^2^C_5_ conformation. The aglycone leaves the catalytic cleft, thus making space for binding of the sialic acid acceptor. Transfer to the acceptor would then occur through attack of the C2 of the sialyl-enzyme intermediate by the 3-OH group of a lactose moiety, or by water (as in other sialidases) which must be deprotonated by the residue acting as acid/base catalyst, Asp59 (Damager et al., 2008). Nevertheless, a mechanism that supports the higher rates for the transfer reaction requires significant conformational changes in the catalytic pocket during substrate binding and catalytic turnover, features not captured in TcTS crystals yet.

Numerous studies that show evidence for the plasticity of the TcTS catalytic cleft have been arisen. For instance, from molecular dynamics simulations it is known that the key hydrophobic residues Y119 and W312 confer flexibility to the catalytic cleft mouth and allow substrates to access the catalytic pocket in a controlled manner (Demir and Roitberg, 2009; Mitchell et al., 2010). Other key residues, which possibly contribute to the plasticity of binding site, were identified by mutagenesis studies (Paris et al., 2001; Carvalho et al., 2010b) or by hybrid quantum mechanics/molecular mechanics simulations (Pierdominici-Sottile and Roitberg, 2011). Crucial evidence for such plasticity rose from the observation that the TS_Y342H_ (Todeschini et al., 2002a) undergoes large conformational changes, upon sialoside binding, leading the overture of a second binding site that accommodates a β-Gal*p* moiety (Todeschini et al., 2004). Active site rearrangement following the sialoside engagement was further proposed for the fully active TcTS (Haselhorst et al., 2004). Nuclear magnetic resonance spectroscopy confirmed that association of the β-Gal*p* within the TcTS active site succeeding the sialic acid donor is necessary for the transfer reaction to proceed (Haselhorst et al., 2004). Results of TS_Y342H_ incubated with α2-6-sialyllactose in the presence of lacto-*N*-tetraose, showing that incorrect fitting of sialoside into the binding site of TS does not trigger β-Gal*p* binding, corroborate this hypothesis. Furthermore, surface plasmon resonance results showing that lactose binds to an inactive mutant of TS_D59N_ in the presence of α2-3-sialyllactose (Buschiazzo et al., 2002).

This discussion shows that further structural data are needed to shed light into the reaction mechanism that underlies efficient sugar transfer activity rather than simple hydrolysis by TcTS. Given that genomic analysis suggests that TcTS proteins have several point mutations (Freitas et al., 2011), structural and mechanistic works must be persistent, as mutations in key amino acids (Paris et al., 2001; Carvalho et al., 2010a) would produce critical modifications in TcTS catalysis and specificity.

## INHIBITION OF SIALIC ACID TRANSFERENCE BY TcTS

Together, the above observations support the hypothesis that TcTS enhances *T. cruzi* virulence by altering host immune responses directed against the parasite. The facts that TcTS presents low homology with mammalian sialidases, and that it is the lonely protagonist for sialic acid acquisition by *T. cruzi*, provide a rationale for a new potential intervention strategy in chemotherapy of Chagas’ disease. Beyond the urgency of alternative drugs to treat the illness, to pursuit of TcTS inhibitors has been the target of several research groups that aim to clarify the role of TcTS in the pathogenesis of Chagas’ disease. However, compounds that effectively inhibit the catalytic activity of TS have not been described. The advances made in this field were somehow indirect, relying on few strategies like the use of neutralizing antibodies, given that the siRNA mechanism of gene silencing in *T. cruzi* is lacking, and TcTS is coded by multiple genes copies (Freitas et al., 2011) making gene deletion experiments unlikely to be successful (Balaña-Fouce and Reguera, 2007).

**FIGURE 5:**
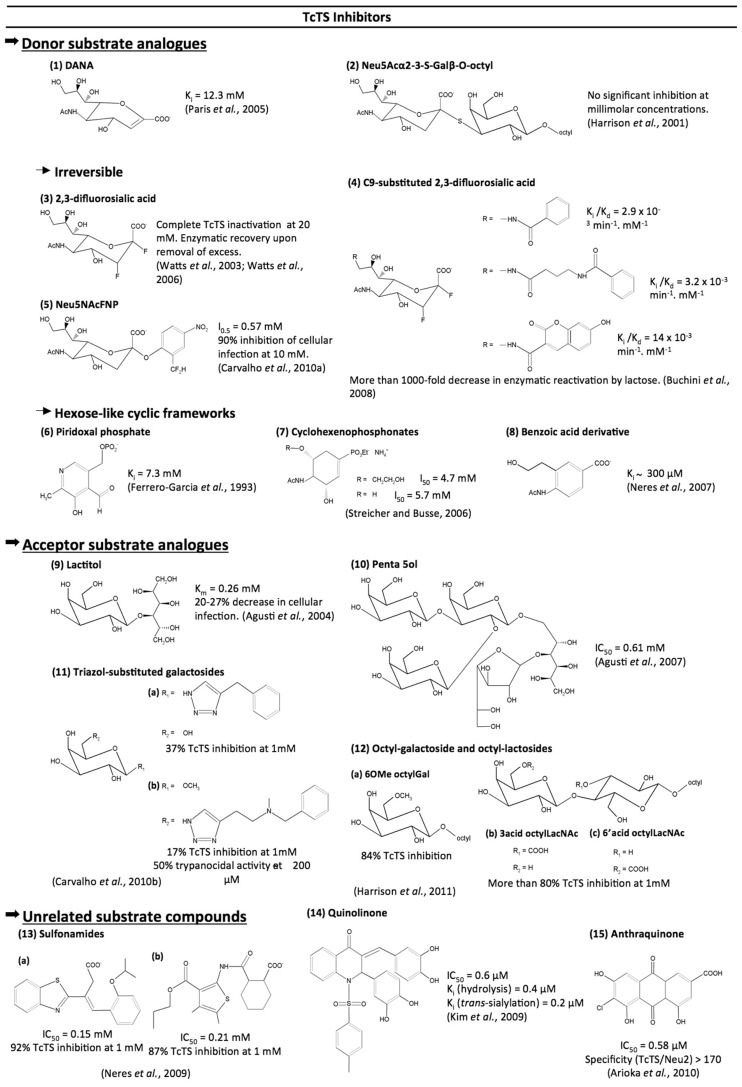
**Compounds tested as TcTS inhibitors**.

Although effective TcTS inhibitors have not yet been reached, efforts made in this area have found interesting lead compounds. The molecules so far tested as TcTS inhibitors are described below as compounds that are analogs to donor or acceptor substrates and compounds that are unrelated to TcTS substrates (**Figure 5**).

### DONOR SUBSTRATE ANALOGS (**Figure 5**)

Unlike the influenza neuraminidase, TcTS activity is barely inhibited by 2-deoxy-2,3-didehydro-*N*-acetylneuraminic acid (DANA), a transition state analog (Paris et al., 2005), or by its analogous 5,6-dihydro-4H-pyran-2-carboxylic acid derivatives including zanamivir (Neres et al., 2006).

Despite the observed success with the use of S-glycosides as inhibitors of glycosidases including sialidases (Watson et al., 2006) the Neu5Acα2-3-S-Galβ-*O*-octyl was found to show no significant inhibition of TcTS even at millimolar concentrations (Harrison et al., 2001). The low inhibition rates observed by the Neu5Acα2-3-S-Galβ-*O*-octyl suggest that the conformations acquired by this compound in solution might not be recognized by the TcTS. This theory can be supported by NMR studies of the conformational distribution of cellobiose and S-cellobiose linked to the *Streptomyces *sp. β-glucosidase*.* These data showed that the S-cellobiose presents three conformational families, unlike cellobiose which is only found in two conformations in solution (Montero et al., 1998).

Trapping of the 2,3-difluorosialic acid by the Tyr342 hydroxyl group opened new avenues for the design of irreversible inhibitors for TcTS (Watts et al., 2003). 2,3-Difluorosialic acid temporarily inactivates the TcTS through covalent binding with the hydroxyl group of Tyr342. However, complete inactivation requires very high concentrations of inhibitor (20 mM) and the enzyme spontaneously recovers its full catalytic activity (Watts et al., 2003). Incorporation of aryl groups at C9, like umbelliferyl, benzamide, and 4-(phenyl carbamide) butyramide, led to more than 1000-fold decrease in TcTS reactivation (Buchini et al., 2008). The crystal of the 9-benzoyl-3-fluoro-*N*-acetylneuraminic acid within TcTS showed that the presence of a voluminous group induced a reorientation of the glycerol side chain, which instead interacts with Tyr119, a site occupied by acceptor substrate, explaining low enzyme reactivation by lactose.

An elegant approach for the irreversible inhibition of TcTS was achieved with the 2-difluoromethyl-4-nitrophenyl-3,5-dideoxy-D-glycero-α-D-galacto-2-nonulopyranosid acid (Neu5AcFNP) and 5-acetamido-2-(4-N-5-dimethylaminonaphthalene-1-sulfo-nyl-2-difluoromethylphenyl)-3,5-dideoxy-D-glycero-α-D-galacto-2-nonulopyranosonic acid (dansyl-Neu5AcFP). Characterization of trapped enzyme by mass spectrometry analysis revealed that inactivation of enzyme occurs through a covalent bond formation between the Arg245 and Asp247 residues with the reactive aglycone generated by the hydrolysis of dansyl-Neu5AcFP. Noteworthy is that Neu5AcFNP inhibited infection of mammalian cells by *T. cruzi* trypomastigotes (Carvalho et al., 2010a).

Apart from complex sugar frameworks, some cyclic scaffolds simulating hexose-like moieties, such cyclohexene, benzoic acid, and pyridine-based structures, were tested. A quite simple pyridoxal phosphate structure was reported as a weak non-competitive TcTS inhibitor (Ferrero-García et al., 1993). The same feature was observed for two *N*-acetyl-clyclohexene phosphonate monoalkyl esters (Streicher and Busse, 2006). Trials using a series of piridine-2-carboxylic acid and benzoic acid derivatives described the 4-acetylamino-3-hydroxymethylbenzoic acid as the best compound, with a Ki value of ~300 µM (Neres et al., 2007). Curiously, this benzoic acid derivative had the same layout as the best cyclohexene phosphonate derivative from the work of Streicher and Busse (2006), evidencing a negative charged group, *N*-acetyl at opposed carbons and a hydroxyl group as pharmacophoric moieties for TcTS inhibition.

### ACCEPTOR SUBSTRATE ANALOGS (**Figure 5**)

In face of fruitless efforts to inhibit TcTS by occupying the donor substrate site, some groups have targeted the acceptor binding site. Lactitol was able to competitively inhibit the TcTS reaction and to interfere with parasite infection in cultured cells (Agusti et al., 2004). Later modifications of the lactitol molecule, by adding Gal*p*, Gal*f*, or benzyl, led to a pentasaccharide with an IC50 of 0.61 mM (Agusti et al., 2007).

The acceptor substrate for the TcTS transfer reaction (Gal*p*) was also used as a source of inspiration for click chemistry synthesis of triazoles-substituted saccharides. Starting from galactose derivatives bearing an azide group at C1 or C6, a triazol-substituted saccharide library was made and tested against TcTS. Despite its low inhibitory activity against TcTS, the *N*-methyl benzylamide derivative presented trypanocidal activity (Carvalho et al., 2010b).

Recently, a series of octyl galactosides and octyl *N*-acetyllactosamines were tested against TcTS. Results showed that the TcTS acceptor binding site is intolerant of substitution of β-Gal*p* at positions 2 and 4, whereas substitution at position 6 of the Gal ring is well accepted, highlighting the potential of 6-substituted Gal residues as TcTS acceptor substrates (Harrison et al., 2011).

### UNRELATED SUBSTRATE COMPOUNDS (**Figure 5**)

The 3-benzothiazol-2-yl-4-phenyl-but-3-enoic acid and sulfonamide scaffolds emerged, from virtual screening, as new frameworks for TcTS inhibition (Neres et al., 2009). Sulfonamides figured also as good substituents for chalcones used as TcTS inhibitors by Kim et al. (2009). Of the compounds tested, the tetrahydroxylated quinolinone inhibits both hydrolytic and TcTS activities at millimolar concentration (Kim et al., 2009). Similar to chalcones, various flavonoids and anthraquinones were systematically screened from a large library. A highly hydroxylated anthraquinone was the best inhibitor of TcTS, with an IC_50_ of 0.58 µM (Arioka et al., 2010). Moreover, this compound did not inhibit Neu2, a mammalian neuraminidase, demonstrating that its inhibition is reasonably specific to TcTS (Arioka et al., 2010). Therefore, this last structure represents, to date, the most promising scaffold for a TcTS inhibitor.

Despite great advances made toward TcTS inhibition, works highlighting the enzyme’s plasticity (Demir and Roitberg, 2009; Mitchell et al., 2010) showing the existence of an acceptor binding site (Todeschini et al., 2004; Damager et al., 2008) suggest that the structure of TcTS with two cavities would be a better framework for rational drug design aimed to TcTS inhibition.

## PERSPECTIVES

Sia participate in a large range of biological processes between *T. cruzi* and its host, and just a few examples are considered here. In fact, the effect of Sia in the pathogenesis of Chagas’ disease remains unknown.

Given the role of Sias as “self-associated molecular patterns” recognized by molecules such as Siglecs (Paulson et al., 2012), TcTS activities can perturb natural self-recognition phenomena, perhaps increasing inflammatory responses by exposing desialylated “danger-associated molecular patterns” (Varki, 2011; Rabinovich et al., 2012).

Moreover, from evidence showing that desialylation of Toll-like receptor 4 (Amith et al., 2010; Abdulkhalek et al., 2011) is essential for receptor activation and cellular signaling, we can speculate that TcTS can also modify host response to “pathogen-associated molecular patterns.”

Recent advances in the glycobiology field will surely help to understand how a glycosidase evolved to have a highly efficient *trans*-glycosidase activity and to put forward the design of specific and potent *TcTS* inhibitors. These molecules will help to disclose the real roles of TcTS/sialoglycoproteins in *T. cruzi* biology, the pathogenesis of Chagas’ disease, and perhaps help the treatment of this illness.

## Conflict of Interest Statement

The authors declare that the research was conducted in the absence of any commercial or financial relationships that could be construed as a potential conflict of interest.

## Acknowledgments

The study was financially supported by Conselho Nacional de Desenvolvimento e Tecnologia (CNPq) Fundaç ã de Amparo à Pesquisa do Estado do Rio de Janeiro, Coordenaç ã de Aperfeiçoamento de Pessoal de Nível Superior and the National Institute for Science and Technology in Vaccines (CNPq 573547/2008-4). The authors wish to thank Leela Davies for her thoughtful comments on this manuscript.
